# Strategies for Optimizing the Supply of N95 Filtering Facepiece Respirators During the Coronavirus Disease 2019 (COVID-19) Pandemic

**DOI:** 10.1017/dmp.2020.160

**Published:** 2020-05-19

**Authors:** Marie A. de Perio, Chad H. Dowell, Lisa J. Delaney, Lewis J. Radonovich, David T. Kuhar, Neil Gupta, Anita Patel, Satish K. Pillai, Maryann D’Alessandro

**Affiliations:** Office of the Director, National Institute for Occupational Safety and Health, Centers for Disease Control and Prevention, Cincinnati, OH; Emergency Preparedness and Response Office, National Institute for Occupational Safety and Health, Centers for Disease Control and Prevention, Atlanta, GA; National Personal Protective Technology Laboratory, National Institute for Occupational Safety and Health, Centers for Disease Control and Prevention, Pittsburgh, PA; Division of Healthcare Quality Promotion, National Center for Emerging and Zoonotic Infectious Diseases, Centers for Disease Control and Prevention, Atlanta, GA; Division of Viral Hepatitis, National Center for HIV/AIDS, Viral Hepatitis, STD, and TB Prevention, Centers for Disease Control and Prevention, Atlanta, GA; Influenza Coordination Unit, National Center for Immunization and Respiratory Diseases, Centers for Disease Control and Prevention, Atlanta, GA; Division of Preparedness and Emerging Infections, National Center for Emerging and Zoonotic Infectious Diseases, Centers for Disease Control and Prevention, Atlanta, GA

**Keywords:** emergency preparedness, infection control, occupational health, pandemics, surge capacity

## Abstract

N95 respirators are personal protective equipment most often used to control exposures to infections transmitted via the airborne route. Supplies of N95 respirators can become depleted during pandemics or when otherwise in high demand. In this paper, we offer strategies for optimizing supplies of N95 respirators in health care settings while maximizing the level of protection offered to health care personnel when there is limited supply in the United States during the 2019 coronavirus disease pandemic. The strategies are intended for use by professionals who manage respiratory protection programs, occupational health services, and infection prevention programs in health care facilities to protect health care personnel from job-related risks of exposure to infectious respiratory illnesses. Consultation with federal, state, and local public health officials is also important. We use the framework of surge capacity and the occupational health and safety hierarchy of controls approach to discuss specific engineering control, administrative control, and personal protective equipment measures that may help in optimizing N95 respirator supplies.

N95 filtering facepiece respirators (commonly called *N95 respirators*) are disposable, tight-fitting air purifying respirators that have a filter efficiency of 95% or greater for a standard test aerosol.^1^ N95 respirators are integral to the personal protective equipment (PPE) most often used to control exposures to infections transmitted via the airborne route in health care settings. Supplies of N95 respirators can become depleted during pandemics or when otherwise in high demand.^2-7^

A number of federal agencies, including the Centers for Disease Control and Prevention (CDC), National Institute for Occupational Safety and Health (NIOSH), Occupational Safety and Health Administration (OSHA), and Food and Drug Administration (FDA), provide standards, guidance, and recommendations for the use of respiratory protection in health care. NIOSH certifies respirators, and the CDC (including NIOSH) develops recommendations on the use of respiratory protection to reduce the transmission of disease in health care settings. OSHA develops and enforces workplace regulations on respiratory protection. The FDA clears the sale of certain types of respirators as medical devices.

The CDC guidance recommends that health care personnel (HCP) follow standard and transmission-based precautions and use N95 or higher level respirators, along with gowns, eye protection (ie, goggles or face shield), and gloves, when entering the room of a patient with confirmed or suspected coronavirus disease 2019 (COVID-19).^8^ In this paper, we offer strategies for optimizing supplies of N95 respirators in health care settings while maximizing the level of protection offered to HCP when there is limited supply in the United States during the COVID-19 pandemic. The strategies are intended for use by professionals who manage respiratory protection programs, occupational health services, and infection prevention programs in health care facilities. Consultation with federal, state, and local public health officials is also important. An accompanying summary list in an easy-to-reference format can be used by health care facilities.^9^

Controlling exposures to occupational hazards is a fundamental way to protect personnel. Traditionally, a hierarchy approach has been used to achieve feasible and optimal control.^10^ Some control measures may fall into multiple categories; multiple strategies can be implemented concurrently and/or sequentially. This hierarchy can be represented as follows:

1. Elimination
2. Substitution
3. Engineering controls
4. Administrative controls
5. PPE

To prevent infectious disease transmission, elimination (physically removing the hazard) and substitution (replacing the hazard) are not typically options for health care settings. However, exposures to transmissible respiratory pathogens in health care facilities can often be reduced or possibly avoided through engineering and administrative controls, which include safe work practices, and PPE.^11,12^

The optimal way to prevent airborne transmission is to use a combination of interventions from across the hierarchy of controls, not just PPE alone. Many of these strategies have been used in experiences with pandemic influenza, measles, tuberculosis, and severe acute respiratory syndrome (SARS).^12-15^ Respirators, when required to protect HCP from airborne contaminants, such as some infectious agents, must be used in the context of a respiratory protection program that meets the requirements of the OSHA Respiratory Protection Standard.^16^ The program must include medical evaluations, training, and fit testing. The program should also include provisions for the cleaning, disinfecting, inspection, repair, and storage of respirators used by HCP on the job according to manufacturer’s instructions.^16^ Proper storage conditions can maximize the shelf life of respirators.

Surge capacity refers to the ability to manage a sudden, unexpected increase in patient volume that would otherwise severely challenge or exceed the present capacity of a facility.^17^ While there are no commonly accepted measurements or triggers to distinguish surge capacity from daily patient care capacity, surge capacity is a useful framework to approach a decreased supply of N95 respirators during the COVID-19 pandemic.

Three general strata have been used to describe surge capacity and can be used to prioritize measures to conserve N95 respirator supplies along the continuum of care.^17^

- Conventional capacity: measures consist of providing patient care without any change in daily contemporary practices. This set of measures should already be implemented in general infection prevention and control plans in health care settings.
- Contingency capacity: measures may change daily standard practices but may not have any significant impact on the care delivered to the patient or the safety of HCP. These practices may be used temporarily during periods of expected N95 respirator shortages.
- Crisis capacity: measures that are not commensurate with contemporary US standards of care. These measures may need to be considered during periods of known N95 respirator shortages.

## CONVENTIONAL CAPACITY STRATEGIES

### Engineering Controls

Engineering controls reduce exposures for HCP by placing a barrier between the hazard and HCP.^10^ Engineering controls can be very effective as part of a suite of strategies to protect HCP without placing primary responsibility of implementation on HCP (ie, they function without HCP having to take an action).

#### Selective Use of Airborne Infection Isolation Rooms

Aerosol-generating procedures performed on patients with confirmed or suspected COVID-19 should take place in an airborne infection isolation room (AIIR). The AIIR should be constructed and maintained in accordance with current guidelines, as recommended in the CDC COVID-19 interim prevention and control recommendations in health care settings.^8^ Air from these rooms should be exhausted directly to the outside or be filtered through a high-efficiency particulate arrestance (HEPA) filter directly before recirculation.^8,18^ Re-circulated air should not be emptied into areas where HCP, visitors, or other people congregate or pass through, such as break areas or thoroughfares.

#### Use of Physical Barriers

Barriers, such as glass or plastic windows, can be an effective solution for reducing exposures among HCP to potentially infectious patients. This approach can be effective in reception areas, such as the desk at the emergency department, triage station, information booth, or pharmacy drop-off/pick-up windows where patients may first report upon arrival to a health care facility. Other examples include the use of curtains between patients in shared areas and closed suctioning systems for airway suctioning for intubated patients.

#### Properly Maintained Ventilation Systems

Another cornerstone of engineering controls is ventilation systems that provide air movement from a clean (HCP workstation or area) to contaminated (sick patient) flow direction (along with appropriate filtration, exchange rate) that are installed and properly maintained.^13^

### Administrative Controls

The term *administrative controls* refers to employer-dictated work practices and policies that reduce or prevent hazardous exposures.^10^ Their effectiveness depends on employer commitment and HCP acceptance and consistent use of the strategies. Regular training, monitoring, and reinforcement are necessary to ensure that policies and procedures are followed consistently.

#### Limit the Number of Patients Going to a Hospital or Outpatient Settings and/or Implement Telemedicine

Health care facilities should consider developing mechanisms to screen patients for acute respiratory illness prior to their health care visits, such as through the appointment reminder system. Postpone and reschedule those with signs and symptoms presenting for non-acute visits. This measure has been helpful during seasonal influenza seasons and during the SARS experience.^19,20^ Nurse advice lines and telemedicine can screen and manage patients with confirmed or suspected COVID-19 without the need for HCP to use N95 respirators. Promoting the use of these technologies and referral networks can help triage persons to the appropriate level of care, potentially reducing the influx of patients to health care facilities seeking evaluation and reserving PPE for when it is needed.

#### Source Control

Health care facilities should identify and assess patients who may be ill with or who may have been exposed to a patient with known COVID-19.^8^ Patients with symptoms of COVID-19 or other respiratory infections, such as fever or cough, presenting for care should use face masks for source control until they can be placed in an AIIR or a private room.^21^ Instructions should include how to use face masks. Patients with these symptoms should not use N95 respirators. If these patients need to leave their room for services in other areas of the hospital (eg, radiology), they should also wear face masks.

#### Exclude HCP Not Directly Involved in Patient Care

Infection prevention and control plans should limit the number of HCP who enter the patient’s room to only those providing direct patient care. Implementation of staffing policies to minimize the number of HCP who enter the room and considerations to exclude staff, such as dietary and housekeeping employees, may extend the supply of N95 respirators.^20^ Exclusion of non-clinical HCP was used by health care facilities caring for patients with Ebola virus disease in the United States.^22^ Similarly, efforts should be made to minimize the number of HCP who are present during an aerosol-generating procedure.

#### Limit Face-to-Face HCP Encounters With Patients With Confirmed or Suspected COVID-19

Measures can be explored to limit face-to-face encounters between HCP and patients with confirmed or suspected COVID-19. HCP may consider bundling care activities to minimize room entries, and bundling may occur across HCP types, such as delivering food trays by HCP when performing other care. Alternative mechanisms for HCP and patient interactions include telephones, video monitoring, and video-call applications on cell phones or tablets.^20,22^

#### Exclude Visitors to Patients With Confirmed or Suspected COVID-19

Restricting visitors from entering the room of a patient with confirmed or suspected COVID-19 is recommended in the CDC COVID-19 interim infection prevention and control recommendations in health care settings.^8^ Alternative mechanisms for patient and visitor interactions, such as video-call applications on cell phones or tablets, should be explored.^22^ Facilities can consider exceptions based on end-of-life situations or when a visitor is essential for the patient’s emotional well-being and care. If visitors must enter the room of a known or suspected COVID-19 patient, facilities should provide instruction (before visitors enter patients’ rooms) on the use of PPE according to current facility policy while in the patient’s room.

#### Cohorting Patients

Cohorting is the practice of grouping together patients who are infected with the same organism to confine their care to 1 area and prevent contact with other patients. Cohorts are created based on clinical diagnosis, microbiologic confirmation when available, epidemiology, and mode of transmission of the infectious agent.^23^ Cohorting has been used extensively for managing outbreaks of multidrug-resistant organisms, including methicillin-resistant *Staphylococcus aureus* (MRSA), vancomycin-resistant enterococci (VRE), multidrug resistant, extended spectrum β-lactamase (ESBL)-producing organisms, *Pseudomonas aeruginosa*; methicillin-susceptible *S. aureus*, respiratory syncytial virus (RSV), adenovirus keratoconjunctivitis, rotavirus, and SARS.^23-25^ When single-patient rooms are not available, patients with **confirmed** COVID-19 may be placed in the same room. Cohorting patients could minimize respirator use when extended use of N95 respirators is implemented.

#### Cohorting HCP

Assigning designated teams of HCP to provide care for all patients with suspected or confirmed COVID-19 could minimize respirator use when extended use is implemented. This strategy, which has been used for tuberculosis, can also limit the number of HCP exposed to patients with COVID-19 and limit the number of HCP who need to be fit tested.^13^

#### Training on Use of N95 Respirators

The OSHA Respiratory Protection Standard requires employers to provide respirator training prior to requiring an employee to use a respirator in the workplace.^16^ Training employees on the proper use of respirators they are expecting to use at work, including putting on and removing them, limitations on their use, and maintenance, is essential for effective use of respiratory protection. Following these measures may help minimize waste of N95 respirators. It is also important that HCP be educated on the use of N95 respirators when caring for patients managed with airborne precautions and other instances for respirator use, such as the performance of aerosol-generating procedures.^16^

#### Just-in-Time Fit Testing

Just-in-time fit testing refers to the capacity of health care facilities to do larger scale evaluation, training, and fit testing of employees when necessary during a pandemic. Facilities may adopt a plan to use the just-in-time method, which has been incorporated into pandemic plans for many facilities.^26^ For large facilities, it may not be feasible to fit test all employees, especially if their job does not typically place them at risk for exposure to airborne infectious diseases, such as tuberculosis. Just-in-time fit testing allows for estimating and optimizing the size of the respiratory protection program and the number of HCP, which can conserve N95 respirators used in training and fit testing.^27^ If health care facilities are expecting to receive COVID-19 patients, they should begin training and start to plan for fit testing prior to receiving patients.

#### Limiting Respirators During Training

The steps of training and fit testing of HCP may be combined into 1 step. If training and fit testing are conducted during 2 separate steps, it may be possible to allow limited reuse of N95 respirators used by individual HCP during fit testing and then training. The respirator might also be saved and then used for patient care.

#### Qualitative Fit Testing

Respirator fit test methods are classified as either qualitative or quantitative, and there are multiple protocols of each classification that are NIOSH-recommended or meet the requirements of the OSHA Respiratory Protection Standard.^16^ A qualitative fit test is a pass/fail test to assess the adequacy of respirator fit that relies on the individual’s sensory detection of a test agent. A quantitative fit test numerically measures the effectiveness of the respirator to seal with the wearer’s face, without relying on the wearer’s voluntary or involuntary response to a test agent. Quantitative fit tests involve adaptation of the respirator to the fit testing equipment, which can involve making holes in the respirator.

Many health care systems already use qualitative fit test methods for fit testing HCP. For those using quantitative fit test methods, considerations can be made to use qualitative fit test methods to minimize the destruction of an N95 respirator used in fit testing and allow for the reuse of the same N95 respirator by HCP. In March 2020, OSHA recommended that health care employers consider changing from a quantitative fit testing method to a qualitative fit testing method.^28^ Qualitative fit testing methods may also allow for rapid fit testing of larger numbers of HCP. While both types of fit tests are considered adequate, there is evidence that quantitative fit tests may be more accurate.^29,30^ Any switch in methods should be assessed to ensure proficiency of the fit testers in carrying out the test.

### Personal Protective Equipment: Respiratory Protection

While engineering and administrative controls should be considered first when selecting controls, the use of PPE should also be part of a suite of strategies used to protect HCP.

#### Surgical N95 Respirators

N95 respirators include standard and surgical N95 respirators. In the United States, all N95 respirators used in occupational settings are approved by NIOSH and used in accordance with OSHA standards. A surgical N95 respirator is a NIOSH-approved N95 filtering facepiece respirator that has also been cleared by the FDA as a surgical mask. Surgical N95 respirators are recommended only for use by HCP who need protection from both airborne and fluid hazards (eg, splashes, sprays). These respirators are not used or needed outside of health care settings. In times of shortages, only HCP who are working in a sterile field or who may be exposed to high velocity splashes, sprays, or splatters of blood or body fluids should be provided these respirators. Other HCP can use standard N95 respirators. If surgical N95 respirators are not available, and there is a risk that the worker may be exposed to high-velocity splashes, sprays, or splatters of blood or body fluids, then a face shield should be worn over the standard N95 respirator.

#### Use of Alternatives to N95 Respirators

Only NIOSH-certified respirators are acceptable to provide appropriate respiratory protection under the OSHA Respiratory Protection Standard, in protecting workers, including HCP from the transmission of airborne infectious disease.^16^ Health care facilities can consider use of NIOSH-approved alternatives to N95 respirators that provide equivalent or higher respiratory protection where feasible.^7^ NIOSH approves other disposable filtering facepiece respirators that are at least as protective as the N95. These include N99, N100, P95, P99, P100, R95, R99, and R100.^31,32^ The N, R, and P designations refer to the filter’s oil resistance. Many filtering facepiece respirators have exhalation valves and should not be used in surgical settings, as unfiltered exhaled breath could compromise the sterile field. On March 2, 2020, the FDA issued an Emergency Use Authorization (EUA) authorizing the use of some NIOSH-approved respirator models in health care settings, including NIOSH-approved filtering facepiece respirators that are not classified as medical devices.^33^

Elastomeric respirators are half-facepiece, tight-fitting respirators that are made of synthetic or rubber material permitting them to be repeatedly disinfected, cleaned, and reused.^27,33,34^ They are equipped with replaceable filter cartridges. Similar to N95 respirators, elastomeric respirators require annual fit-testing but they are not destroyed during testing. Similar to filtering facepiece respirators with exhalation valves, elastomeric respirators should not be used in surgical settings due to concerns that exhaled breath of the wearer is not filtered when coming out of the exhalation valve and may contaminate the sterile field.

Powered air purifying respirators (PAPRs) are reusable respirators that are typically loose-fitting hoods or helmets but can also be tight-fitting. These respirators are battery-powered with a blower that pulls air through attached filters or cartridges. There are 3 PAPR filter classes. These include high efficiency (HE) filters, PAPR 100-N filters, and PAPR 100-P filters.^31^ Loose-fitting PAPRs do not require fit-testing and can be used with facial hair, unlike tight-fitting respirators. However, PAPRs should not be used in surgical settings due to concerns that the blower exhaust and exhaled air may contaminate the sterile field. Facilities using elastomeric respirators and PAPRs should have up-to-date cleaning/disinfection procedures.^35^

On March 28, 2020, the FDA issued an update to address NIOSH-approved air purifying respirators for use in health care settings during response to the COVID-19 public health emergency.^33^

## CONTINGENCY CAPACITY STRATEGIES

These contingency capacity strategies accompany and build on the conventional capacity strategies.^17^

Decisions to implement contingency and crisis strategies^17^ are based upon these assumptions:

1. Facilities understand their current N95 respirator inventory and supply chain.
2. Facilities understand their N95 respirator utilization rate.
3. Facilities are in communication with local health care coalitions, federal, state, and local public health partners (eg, public health emergency preparedness and response staff) regarding identification of additional supplies.
4. Facilities have already implemented all or many conventional capacity measures.
5. Facilities have provided HCP with required education and training, including having them demonstrate competency with donning and doffing with any PPE ensemble that is used to perform job responsibilities, such as provision of patient care.

### Administrative Controls

#### Decrease Length of Hospital Stay for Medically Stable Patients With COVID-19

The CDC recommends discharging patients with confirmed COVID-19 when they are medically stable and have an appropriate home environment to which to return.^36^ If patients cannot be discharged to home for social rather than medical reasons, public health officials might need to identify alternative, non-hospital housing where those patients can convalesce.

#### Temporarily Suspend Annual Fit Testing

In March 2020, OSHA issued new temporary guidance regarding the enforcement of the OSHA Respiratory Protection Standard.^16,28^ The guidance gave OSHA field offices enforcement discretion concerning the annual fit testing requirement as long as HCP have undergone an initial fit test with the same model, style, and size. Other conditions included that employers have made a good-faith effort to comply with the standard and that only NIOSH-certified respirators are used. The guidance emphasized the need to explain to HCP the importance of conducting a seal check each time the respirator is put on and conducting a fit test if there are visual changes to the employee’s physical condition.^16,28^

### Personal Protective Equipment: Respiratory Protection

#### Use of N95 Respirators Beyond the Manufacturer-Designated Shelf Life for Training and Fit Testing

In times of shortage, consideration can be made to use N95 respirators beyond the manufacturer-designated shelf life. However, these respirators might not perform to the requirements for which they were certified. Over time, components such as the strap and filter material may degrade, which can affect tension and the quality of the fit and seal.^37^ Because of this, use of expired respirators could be prioritized for situations where HCP are NOT exposed to pathogens, such as during training and fit testing. As expired respirators can still serve an important purpose, health care facilities should retain all N95 respirators during the outbreak.

#### Extended Use of N95 Respirators

Extended use refers to the practice of wearing the same N95 respirator for repeated close contact encounters with several different patients, without removing the respirator between patient encounters.^38,39^ The decision to implement policies that permit extended use of N95 respirators should be made by the professionals who manage the institution’s respiratory protection program, in consultation with their occupational health and infection control departments with input from the state/local public health departments. The CDC has recommended guidance on implementation of extended use of N95 respirators in health care settings.^38,39^ Extended use has been recommended and used as an option for conserving respirators during previous respiratory pathogen outbreaks and pandemics.^38,39^

Extended use is well suited to situations wherein multiple patients with the same infectious disease diagnosis, whose care requires use of a respirator, are cohorted (eg, housed on the same hospital unit).^38^ It can also be considered to be used for care of patients with tuberculosis, varicella, and measles, other infectious diseases where use of an N95 respirator or higher is recommended. When practicing extended use of N95 respirators, the maximum recommended extended use period is 8–12 hours.^38^ Respirators should not be worn for multiple work shifts and should not be reused after extended use. N95 respirators should be removed (doffed) and discarded before activities, such as meals and restroom breaks. N95 respirators should also be removed and discarded if soiled, damaged, or hard to breathe through.

## CRISIS CAPACITY STRATEGIES

These crisis capacity strategies accompany and build on the conventional and contingency capacity strategies.^17^

### When N95 Supplies Are Running Low: Personal Protective Equipment – Respiratory Protection and Face Masks

#### Use of Respirators Beyond the Manufacturer-Designated Shelf Life for Health Care Delivery

Consideration can be made to use N95 respirators beyond the manufacturer-designated shelf life for care of patients with COVID-19, tuberculosis, measles, and varicella. As discussed previously, respirators beyond the manufacturer-designated shelf life may not perform to the requirements for which they were certified. A recent study of filtering facepiece respirators beyond their manufacturer-designated shelf life that were stockpiled throughout the country found that 98% of the models evaluated continued to perform in accordance with NIOSH standards.^40^ Some models continued to meet the requirements of the approval and should perform as expected, provided a visual inspection shows that characteristics, such as the strap and nose bridge material, have not degraded. The CDC/NIOSH issued guidance to provide stockpile managers information needed to make decisions regarding the release of stockpiled respirators to support the COVID-19 response.^41^ These respirators should be used in the context of a respiratory protection program that includes medical evaluation, training, and fit testing.^16^ If used in health care delivery, it is particularly important that HCP perform the expected seal check, prior to entering a patient care area. On March 2, 2020, the FDA issued an EUA authorizing the use of certain NIOSH-approved respirator models in health care settings. This EUA includes respirator units that are past their designated shelf life.^33^ In April 2020, OSHA issued additional temporary guidance giving OSHA field offices enforcement discretion for the use of N95 respirators beyond the manufacturer’s recommended shelf life.^42^

#### Use of Respirators Approved Under Standards Used in Other Countries or Jurisdictions That are Similar to NIOSH-Approved N95 Respirators

Other countries approve respirators for occupational use according to country-specific standards.^43^ These products are evaluated using some methods that are similar to those used by NIOSH. Some methods are different but are expected to provide protection similar to NIOSH-approved filtering facepiece and elastomeric respirators. Devices supplied by current NIOSH-approval holders producing respirators under the standards authorized in the listed countries are expected to provide the protection indicated, given that a proper fit is achieved. Therefore, they are generally considered to be suitable alternatives to provide protection during the COVID-19 response when supplies are short. Within Tables 1 and 2, the country, conformity assessment standards, standards and guidance documents, acceptable product classification, and NIOSH classification are provided in alphabetical order. All of these respirators have protection factors of at least 10 in the countries listed, as outlined in the standards and guidance documents specified.

**TABLE 1:** Respirators Approved Under Standards Used in Other Countries That Are Similar to NIOSH-Approved N95 Filtering Facepiece Respirators

Country	Performance Standard	Acceptable Product Classification	May Be Used in Lieu of NIOSH-Certified Products Classified as:
Australia	AS/NZS 1716:2012	P2	N95
		P3	N99 or lower
Brazil	ABNT/NBR 13698:2011	PFF2	N95
		PFF3	N99 or lower
People’s Republic of China	GB 2626-2006 GB 2626-2019 GB 19083-2010	KN/KP95	N95
		KN/KP100	N95
Europe	EN 149-2001	P2	N95
		P3	N99 or lower
Japan	JMHLW-2000	DS/DL2	N95
		DS/DL3	N99 or lower
Korea	KMOEL-2017-64	Special 1st	N95
Mexico	NOM-116-2009	N95	N95
		R95	R95 or lower
		P95	P95 or lower
		N99	N99 or lower
		R99	R99 or lower
		P99	P99 or lower
		N100	N100 or lower
		R100	R100 or lower
		P100	P100 or lower

**TABLE 2:** Respirator-Cartridge Units Approved Under Standards Used in Other Countries That Are Similar to NIOSH-Approved Elastomeric Half-Facepiece Respirators

Country	Performance Standard	Acceptable Product Classification	May Be Used in Lieu of NIOSH-Certified Products Classified as:
Australia	AS/NZS 1716:2012	P2	N95
		P3	N99 or lower
Brazil	ABNT/NBR 13694:1996; ABNT/NBR 13697:1996	P2	N95
		P3	N99 or lower
People’s Republic of China	GB 2626-2006;	KN/KP95	N95
	GB 2626-2019 GB 19083-2010	KN/KP100	N95
Europe	EN140-1999; EN 143-2000	P2	N95
		P3	N99 or lower
Japan	JMHLW-2000	RS/RL2	N95
		RS/RL3	N99 or lower
Korea	KMOEL-2014-46	Special 1st	N95
Mexico	NOM-116-2009	N95	N95
		R95	R95 or lower
		P95	P95 or lower
		N99	N99 or lower
		R99	R99 or lower
		P99	P99 or lower
		N100	N100 or lower
		R100	R100 or lower
		P100	P100 or lower

Non-NIOSH-approved products developed by manufacturers who are not NIOSH approval holders are expected to meet the performance requirements if they have been issued a certificate of approval by an authorized test laboratory indicating they conform to the standards identified in Tables 1 and 2. On March 28, 2020, the FDA issued an EUA regarding non-NIOSH-approved disposable filtering facepiece respirators imported from Australia, Brazil, Europe, Japan, Korea, or Mexico.^44^ On May 7, 2020, FDA issued an update to the Non-NIOSH-approved respirator EUA concerning non-NIOSH-approved respirators that have been imported from China.^45^ In April 2020, OSHA issued additional temporary guidance giving OSHA field offices enforcement discretion for the use of N95 respirators that comply with standards of other countries.^46^ Non-NIOSH-approved products developed by manufacturers who are not NIOSH approval holders (and do not have a certificate of approval from an authorized test laboratory from 1 of the countries identified within the FDA EUA) should only be used in crisis situations when no NIOSH-approved N95 respirator (or a listed device within the FDA EUA) is available. These devices should not be used during aerosol-generating medical procedures unless the alternative is a loose-fitting surgical mask or improvised device. CDC guidance is available to provide users with instructive information about counterfeit respirators, substandard respirators, and factors to consider when planning to purchase non-NIOSH-approved respirators from another country.^47-49^

#### Limited Reuse of N95 Respirators

Reuse refers to the practice of using the same N95 respirator by 1 HCP for multiple encounters with different patients but removing it (ie, doffing) after each encounter.^38,39^ This practice is often referred to as *limited reuse* because restrictions are in place to limit the number of times that the same respirator is reused. It is important to consult with the respirator manufacturer regarding the maximum number of donnings or uses they recommend for the N95 respirator model. If no manufacturer guidance is available, data suggest limiting the number of reuses to no more than 5 uses per device to ensure an adequate safety margin.^50^ N95 and other disposable respirators should not be shared by multiple HCP. The CDC has guidance on the implementation of limited reuse of N95 respirators in health care settings.^38,39^

For pathogens for which contact transmission is not a concern, routine limited reuse of single-use disposable respirators has been practiced for decades. For example, for tuberculosis prevention, a respirator classified as disposable can be reused by the same provider as long as the respirator maintains its structural and functional integrity.^12,51,52^ If reuse must be implemented in times of shortages, HCP could be encouraged to reuse their N95 respirators when caring for patients with tuberculosis disease first.

Limited reuse of N95 respirators when caring for patients with COVID-19 might also become necessary. However, it is unknown what the potential contribution of contact transmission is for SARS-CoV-2 (the virus that causes COVID-19), and caution should be used.^53,54^ The surfaces of a NIOSH-approved N95 respirator will become contaminated with pathogens while filtering the inhalation air of the wearer during exposures to pathogen laden aerosols.^50,51^ The pathogens on the filter materials of the respirator may be transferred to the wearer upon contact with the respirator during activities, such as adjusting the respirator, improper doffing of the respirator, or when performing a user-seal check when redonning a previously worn respirator. Reuse has been recommended as an option for conserving respirators during previous respiratory pathogen outbreaks and pandemics. It may also be necessary to reuse N95 respirators when caring for patients with varicella or measles, although contact transmission poses a risk to HCP who implement this practice. Ideally, N95 respirators should not be reused by HCP who care for patients with COVID-19 then care for other patients with varicella, measles, and tuberculosis, and viceversa.

Respirators grossly contaminated with blood, respiratory or nasal secretions, or other bodily fluids from patients should not be reused. HCP can consider using a face shield or face mask over the respirator to reduce/prevent contamination of the N95 respirator. HCP reusing an N95 respirator should use a clean pair of gloves when donning or adjusting a previously worn N95 respirator. It is important to discard gloves and perform good hand hygiene after the N95 respirator is donned or adjusted.

One effective strategy to mitigate the contact transfer of pathogens from the respirator to the wearer could be to issue each HCP who may be exposed to COVID-19 patients a minimum of 5 respirators. Each respirator will be used on a particular day and stored in a breathable paper bag or cardboard box until the next week. This will result in each worker requiring a minimum of 5 N95 respirators if they put on, take off, care for them, and store them properly each day. This amount of time in between uses of each respirator should exceed the 72-hour expected survival time for SARS-CoV-2.^55^ HCP should still treat the respirator as though it is still contaminated and follow the precautions outlined in the CDC reuse recommendations.^38^

Respirator manufacturers may provide guidance for respirator decontamination. At present, there are no generally approved methods for respirator decontamination prior to reuse. Decontamination might cause poorer fit, filtration efficiency, and breathability of disposable filtering facepiece respirators as a result of changes to the filtering material, straps, nose bridge material, or strap attachments of the filtering facepiece respirator. Based on the limited research available, ultraviolet germicidal irradiation, vaporous hydrogen peroxide, and moist heat have shown the most promise as potential methods to decontaminate filtering facepiece respirators.^56^

#### Use of Additional Respirators Beyond the Manufacturer-Designated Shelf Life for Health Care Delivery That Have Not Been Evaluated by NIOSH

Use of additional N95 respirators beyond the manufacturer-designated shelf life for care of patients with COVID-19, tuberculosis, measles, and varicella can be considered. Some models found in stockpiles have been found NOT to perform in accordance with NIOSH performances standards, and other models have not been evaluated by NIOSH.^40,41^ Consideration can be given to use these N95 respirators beyond the manufacturer-designated shelf life. These respirators should ideally be used in the context of a respiratory protection program that includes medical evaluation, training, and fit testing.^16^ It is particularly important that HCP perform a user seal check, prior to entering a patient care area.

#### Prioritize the Use of N95 Respirators and Face Masks by Activity Type

This prioritization approach to conservation (Table 3) is intended to be used when N95 respirators are so limited that routinely practiced standards of care for all HCP wearing N95 respirators when caring for a COVID-19 patient are no longer possible. The use of N95s or elastomeric respirators or PAPRs should be prioritized for HCP with the highest potential exposures, including being present in the room during aerosol-generating procedures performed on patients with confirmed or suspected COVID-19. When face masks must be used by HCP entering a patient care area, source control by masking patients and maintaining distance from the patient is particularly important to reduce the risk of transmission.^8,20^

**TABLE 3:** Suggested Face Mask or Respirator Use, Based Upon Distance From a Patient With Suspected or Known COVID-19 and Use of Source Control^*^

HCP Planned Proximity to the Case Patient During Encounter	Face Mask or Respirator Determination	
	Patient Masked for Entire Encounter (that is, with source control)	Unmasked Patient or Mask Needs to be Removed for Any Period of Time During the Patient Encounter
HCP will remain at greater than 6 feet from symptomatic patient.	If HCP must enter the patient care area: no face mask or respirator. However, HCP should consider not entering the patient care area.	If HCP must enter the patient care area: no face mask or respirator. However, HCP should consider not entering the patient care area.
HCP will be within 6 feet of symptomatic patient, including providing direct patient care.	Face Mask	Any NIOSH-approved N95 respirator/ elastomeric/PAPR, based on availability or face mask if respirator unavailable.
HCP will be present in the room during aerosol-generating procedures performed on symptomatic persons.	Any NIOSH-approved N95 respirator/ elastomeric/PAPR, based on availability.	Any NIOSH-approved N95 respirator/ elastomeric/PAPR, based on availability.

* Based on availability, organizations may require and/or individuals may voluntarily choose to use higher levels of protection. COVID-19 = 2019 novel coronavirus disease; HCP = health care personnel; PAPR = powered air-purifying respirator.

### When No Respirators Are Left: Administrative Controls

#### Exclude HCP at Higher Risk for Severe Illness From COVID-19 From Contact With Known or Suspected COVID-19 Patients

During severe resource limitations, consider excluding HCP who may be at higher risk for severe illness from COVID-19, such as those of older age, those with chronic medical conditions, and/or those who may be pregnant, from caring for patients with confirmed or suspected COVID-19 infection^57^.

#### Consider Designating Convalescent HCP for Provision of Care to Known or Suspected COVID-19 Patients

It may be possible to designate HCP who have clinically recovered from COVID-19 to provide care for additional patients with COVID-19. Individuals who have recovered from COVID-19 infection may have developed some protective immunity, but this has not yet been proven. For Middle East respiratory syndrome, antibody persistence was found to depend on disease severity.^58^ The antibody responses to SARS-CoV-2 and implications for immunity are not yet fully understood.^59-61^

### Engineering Controls

#### Expedient Patient Isolation Rooms for Risk Reduction

Portable fan devices with HEPA filtration that are carefully placed can increase the effective air changes per hour of clean air to the patient room, reducing risk to individuals entering the room without respiratory protection. Portable HEPA filtration systems can be used to create expedient patient isolation rooms.^62,63^ This approach involves establishing a high-ventilation-rate, negative pressure, inner isolation zone that sits within a “clean” larger ventilated zone. In the absence of any remaining supply of N95 respirators, it may be possible to use this technology in conjunction with HCP wearing face masks.

#### Ventilated Headboards

NIOSH has developed the ventilated headboard that draws exhaled air from a patient in bed into a HEPA filter, decreasing risk of HCP exposure to patient-generated aerosol.^64^ This technology consists of lightweight, sturdy, and adjustable aluminum framing with a retractable plastic canopy. The ventilated headboard can be deployed in combination with HEPA fan/filter units to provide surge isolation capacity within a variety of environments, from traditional patient rooms to triage stations, and emergency medical shelters. In the absence of any remaining supply of N95 respirators, it may be possible to use this technology in conjunction with HCP and/or patients wearing face masks.

#### HCP Use of Non-NIOSH-Approved Masks

In settings where neither respirators nor face masks are available, as a last resort, it may be necessary for HCP to use masks that have never been evaluated or approved by NIOSH. However, these masks are not considered PPE, since their capability to protect HCP is unknown. Caution should be exercised when considering this option.^65,66^ The FDA has issued guidance on their enforcement policy for face masks.^67^

## CONCLUSION

Many of the engineering control, administrative control, and PPE strategies listed in the conventional capacity strategies should already be implemented by health care facilities as part of general infection prevention plans. These measures may optimize supplies of N95 respirators while maximizing the level of protection offered to HCP in health care settings. The use of NIOSH-approved alternatives to N95 respirators, including other classes of filtering facepiece respirators, elastomeric respirators, and PAPRs, should be considered before considering contingency and crisis capacity strategies. As health care facilities consider implementing contingency capacity and crisis capacity strategies due to shortages experienced during the COVID-19 pandemic, it is important to regularly consult with federal, state, and local public health officials for additional guidance. The crisis capacity strategies provided in this paper are not commensurate with contemporary US standards of care and should be implemented with caution. As N95 respirator availability increases, health care facilities should promptly resume standard practices.

These strategies highlight the importance of future research to determine the relative contributions of various modes of transmission of SARS-CoV-2 to inform recommended PPE. It will also be useful to understand how long SARS-CoV-2 can remain infective in the air and on surfaces of respirators and other PPE. In addition, it is important for future research to evaluate the effectiveness of respirators that (1) are past their intended manufacturer’s shelf life, (2) are worn as part of extended use, (3) comply with international standards, and (4) are decontaminated and reused. Evaluating effectiveness involves evaluating filtration performance and fit performance. It is critical that science continue to inform public health planning and practice to keep HCP healthy and safe during this pandemic.
